# Editorial for Special Issue “Yeast in Winemaking”

**DOI:** 10.3390/microorganisms9050940

**Published:** 2021-04-27

**Authors:** Hervé Alexandre

**Affiliations:** UMRProcédésAlimentaires et Microbiologiques, Equipe VAlMiS (Vin, Aliment, Microbiologie, Stress), AgroSupDijon—Université de Bourgogne/Franche-Comté, IUVV, Rue Claude Ladrey, BP 27877, 21000 Dijon, France; rvalex@u-bourgogne.fr

Yeast in winemaking was first studied for its role in alcoholic fermentation, and has led to the publication of a huge amount of scientific articles. Since then, scientists have tried to understand yeast metabolism in different yeast species, yeast interactions, and aroma production by yeast in order to master the wine profile. This Special Issue has gathered 11 articles reflecting the efforts realized by the scientific community in order to understand how to better control wine quality and wine profile.

Many efforts have been done to identify yeast involved in the wine profile. While DNA based tools allowed precise yeast taxonomy, it has recently been proved the existence of a strong link between physiology and taxonomy suggesting that many loci could be particularly interesting as “double usage” markers for taxonomy and general metabolic evolution or ethanol stress [1]. Besides *Saccharomyces cerevisiae*, from many years now, it has been shown that the so-called ‘non-Saccharomyces’ encompasses different yeast species that contribute positively to wine quality thanks to their flavor contribution. Russo et al. [2] report the impact of *Candida zemplinina* on the final aroma of produced wine. Aging on yeast lees is also a winemaking strategy that impacts wine quality. Autolysis during aging on yeast lees is responsible for the observed changes. A correlation between autophagy and autolysis has been proposed in order to accelerate the acquisition of wine organoleptic properties during sparkling wine elaboration. It has been shown by Porras-Agüera et al. [3] that differences in autophagy-related proteomes between strains and conditions exist. Furthermore, the same authors proposed that the use of flor yeast in sparkling wine production would be interesting considering its fast cell death during the process [4]. Moreover, Gonzalez-Jimenez [5] reports that flor yeast may be proposed for sparkling wine production to enhance the diversity and typicity of sparkling wine yeasts. Wine quality needs to be controlled by preventing the development of spoilage microorganisms. In that sense, a good comprehension of these microorganisms is necessary. An example is given in this Special Issue with Brettanomyces, which is considered the nightmare of winemakers. G-Poblete et al. [6] report that p-coumaric acid has a protective action against the toxic effects of SO_2_.

Scientists should also consider consumer expectation, and, nowadays, a demand for low ethanol wine and low sulfite content wine exists. In this context, strategies are being developed to decrease ethanol [7] and sulfite content in wine [8]. Consumers are also seeking wine fermented with indigenous yeast. The control of such fermentation is more difficult, because a consortium of yeast is present with a specific population dynamic across the alcoholic fermentation. The final results are intimately linked to the interactions between microorganisms. Knowledge regarding such interactions is important to control indigenous fermentation. These interactions exist between yeast and bacteria as reported by du Toit et al. [9] and between yeasts [10].

The yeast consortium has been proposed to play a role to the Terroir effect, however contradictory results has been published, thus, the concept of microbial terroir has been examined [11].

This Special Issue reminds us of the prominent role of yeast at each step of the winemaking process. Indeed, yeast is not only responsible for alcoholic fermentation, but could also be used for different applications, as described in these articles.

## Data Availability

Not applicable.
